# Chemical Space Virtual Screening against Hard-to-Drug RNA Methyltransferases DNMT2 and NSUN6

**DOI:** 10.3390/ijms24076109

**Published:** 2023-03-24

**Authors:** Robert A. Zimmermann, Tim R. Fischer, Marvin Schwickert, Zarina Nidoieva, Tanja Schirmeister, Christian Kersten

**Affiliations:** Institute of Pharmaceutical and Biomedical Sciences, Johannes Gutenberg-University, Staudingerweg 5, 55128 Mainz, Germany

**Keywords:** RNA methyltransferases, DNMT2, NSUN6, virtual screening, ultra-large molecular libraries, molecular docking, chemical spaces

## Abstract

Targeting RNA methyltransferases with small molecules as inhibitors or tool compounds is an emerging field of interest in epitranscriptomics and medicinal chemistry. For two challenging RNA methyltransferases that introduce the 5-methylcytosine (m^5^C) modification in different tRNAs, namely DNMT2 and NSUN6, an ultra-large commercially available chemical space was virtually screened by physicochemical property filtering, molecular docking, and clustering to identify new ligands for those enzymes. Novel chemotypes binding to DNMT2 and NSUN6 with affinities down to K_D,app_ = 37 µM and K_D,app_ = 12 µM, respectively, were identified using a microscale thermophoresis (MST) binding assay. These compounds represent the first molecules with a distinct structure from the cofactor SAM and have the potential to be developed into activity-based probes for these enzymes. Additionally, the challenges and strategies of chemical space docking screens with special emphasis on library focusing and diversification are discussed.

## 1. Introduction

### 1.1. RNA Methyltransferases as a Target

RNA modifications play an important role in an abundance of both physiological and pathophysiological biochemical pathways [1,2,3,4]. Among over 170 known RNA modifications [5,6,7], one of the most significant ones is methylation, which is introduced by methyltransferases. One prominent example of interfering with RNA modifying enzymes as a therapeutic strategy is the methyltransferase 3 (METTL3, also called *N*^6^-adenosine-methyltransferase) inhibitor STM2457, which is under investigation for the treatment of acute myeloid leukemia (AML) [8]. Examination of other RNA methyltransferases as possible drug targets is still in its infancy, which is also reflected in the literature [9]. However, in recent times, research in this area has started to accelerate. In this emerging field, the 5-methylcytosine (m^5^C) modification, which is catalyzed by various members of the Nol1/Nop2/SUN (NSUN) family, but also by the DNA methyltransferase 2 (DNMT2), is of special interest in different human diseases [10].

**Figure 1:**
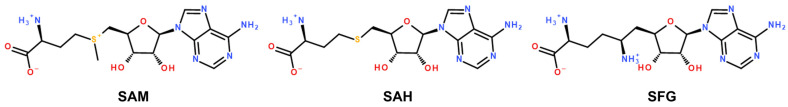
Molecular structures of the DNMT2 and NSUN6 cofactor SAM, the reaction product and auto-inhibitor SAH and the pan-methyltransferase inhibitor SFG.

Due to its high structural similarity to DNMT1 and DNMT3, DNMT2 was classified as a member of the DNMT family, but it was found that the main substrate of DNMT2 is RNA. The first reported RNA substrate of DNMT2 was tRNA^Asp^ [11,12,13]. Meanwhile, tRNA^Val^ and tRNA^Gly^ were identified as substrates of DNMT2 as well [14,15]. The m^5^C modification introduced by DNMT2 in position C-38 of the anticodon loop of tRNA^Asp^ increases the stability of the tRNA and therefore affects protein translation [16,17,18]. The influence of DNMT2 involves epigenetic but also pathogenic pathways, especially in carcinogenesis and inheritance of metabolic disorders [19,20,21]. Besides azacytidine and zebularine, which both have to be incorporated into the substrate tRNA to inhibit DNMT2 [22,23], several derivatives of the enzyme’s cofactor *S*-adenosyl-L-methionine (SAM, Figure 1) and the autoinhibitory reaction product *S*-adenosyl-L-homocysteine (SAH) and the well-known natural product pan-methyltransferase inhibitor sinefungin (SFG) were identified to inhibit DNMT2 [24,25].

NSUN6 is a member of the NSUN family and methylates C-72 in tRNA^Cys^ and tRNA^Thr^, as well as several mRNAs [26,27]. NSUN6 was claimed to be involved in bone metastasis [28], but its complete physiological role remains elusive [29]. Besides SAH, sinefungin, and derivatives, to the best of our knowledge, no drug-like inhibitors designed for NSUN6 have been reported in the literature so far. Although NSUN6 and DNMT2 may not be potential drug targets in the first place, the development of activity-based probes (ABPs) [30,31] for RNA methyltransferases aims to improve our understanding of the biological impact of RNA methylation in general via chemical knock-out in cellular models. Therefore, the requirements for ABPs can be less strict in terms of drug metabolism and pharmacokinetics than for actual drug candidates while still requiring high affinity and selectivity.

### 1.2. Ultra-Large Library Docking

With the advance of commercial, combinatorial make-on-demand chemical spaces, structure-based virtual screening faces new opportunities and challenges. With the knowledge of robust reactions and available building blocks, new molecular entities become available for ultra-large library (also called chemical spaces) virtual screenings while being likely to be synthetically accessible at the same time. These chemical spaces hold the promise that included novel chemotypes can bind to so-far-undrugged targets. Current make-on-demand spaces are far beyond the size of in-stock compounds. While the curated ZINC20 library [32] covers around 8.1 million drug-like [33] in-stock molecules (molecular weight ≤ 500 g/mol, logP ≤ 5, reactivity: anodyne) from a plethora of different supplier catalogs, current commercial chemical spaces overshadow these by three (e.g., WuXi LabNetworks’s GalaXi, 8 × 10^9^ molecules) to almost four orders of magnitude (e.g., Enamine’s REALspace, 3.4 × 10^10^, values from December 2022) [34,35]. While exhaustive molecular docking screens (in the following referred to as ‘brute-force docking’) can be feasible up to many millions to a few billion molecules, they demonstrated impressive hit rates and identified potent binders previously (Table 1) [36,37,38,39], increasing sizes of chemical spaces will make this approach (computationally) too expensive if not impossible [40]. This especially holds true if proprietary chemical spaces are considered, such as Merck MASSIV 2018 (10^20^ molecules) or GSK XXL 2020 (10^26^ molecules) [34,41]. Subsequently, even though ‘bigger is better’ [42] is usually valid for virtual screening libraries, new strategies for structure-based screenings are required to focus libraries prior to docking [43,44]. One approach is the docking of a diverse subset. However, even though this speeds up the docking time, it cannot be known a priori if the diverse cluster representatives are suitable for the target of interest. Eventually, complete clusters of likely binders are discarded if the cluster representative does not match a required interaction pattern [36]. This likewise accounts for random subsets, but in combination with a machine-learning (ML) model to quickly estimate docking scores, this strategy yielded some promising results for speeding up the process while maintaining high hit rates recently [40]. Alternatively, taking advantage of target knowledge can be a promising route to design its own focused chemical spaces, as demonstrated in an exclusive series of tetrahydropyridines as potential serotonin (5-hydroxytryptamine, 5-HT) receptor ligands [45]. Another approach is based on fragment-based drug design (FBDD), either physically by generating a chemical space upon crystallographically known fragment substructures and corresponding building blocks [46,47] or starting with pure fragment docking [48,49]. While both strategies rely on the placement of initial virtual ‘synthons’, a crystallographic fragment screening as a first step can support the docking process using the experimental binding mode for template docking, whereas the latter is defined by general limitations of fragment docking. The limitation that probably requires the most attention in this regard is that scoring functions might be unable to distinguish the correct binding mode from incorrect ones due to the intrinsically low number of interactions of fragments requiring proper additional re-scoring methods or pharmacophore constraints [47,49,50,51].

While for the described virtual screening strategies, several success stories are reported with both high hit rates and very potent ligands (Table 1), those virtual screenings were usually performed for very well-described targets such as kinases and G-protein-coupled receptors (GPCRs) with several crystal structures and known ligands available. However, virtual chemical spaces hold the promise to contain novel chemotypes not (yet) present in conventional in-stock libraries as suitable ligands for so-far-undrugged and considered undruggable or hard-to-drug targets. In our study, we applied the virtual screening strategy on targets of interest DNMT2 and NSUN6 with only a few known ligands, a small number of crystal structures, and rather low predicted druggability, where hit rates tend to be lower (Table 1) [52,53,54,55]. Reported ligands for DNMT2 and NSUN6 are either close homologs of the native cofactor SAM with poor physicochemical properties, low drug-likeness, and limited selectivity over other SAM-dependent enzymes [25] or require incorporation into the substrate tRNA-like 5-azacytidine [23]. Likewise, the drug-candidate inhibitor of the structurally closely related DNMT1, GSK3685032, was recently shown to bind primarily to the DNA rather than to the enzyme [56]. This molecule was optimized from only one hit series of a 1.8 million compound high-throughput screening (HTS; most other initial hits were not followed up due to inactivity after purification or non-specific binding). This is a further hint for the low druggability of DNMTs, which eventually requires novel chemotypes to identify new ligands. As another consequence of the low number of known ligands for the m^5^C-RNA methyltransferases of interest, also model validation is considered best practice [37], which includes binder vs. non-binder/decoy discrimination offered limited possibilities.

**Table 1:** Recent examples of (ultra-)large library structure-based virtual screenings and results from this work.

Target	Reported Ligands ^a^	PDB-Entries ^b^	PDB-ID	Predicted Druggability ^c^	VS Strategy	VS Library Size	Synthesis Success Rate	Hit Rate	Most Potent Hit (→ Improved Lead Compound)	References
D_4_	4457	96	5WIU	0.74	Brute force	138 million	549/589 (93%)	58/238 (24%)	EC_50_ = 180 pM	[36]
AmpC	62,046	123	1L2S	0.40	Brute force	99 million	44/51 (86%)	5/44 (11%)	K_I_ = 1.3 µM	[36]
MT_1_	1334 (MT_1A_)	12	6ME3	0.67	Brute force	150 million	38/40 (95%)	15/38 (39%)	EC_50_ = 470 pM	[38]
KEAP1	704 (KEAP1/ NRF2)	125	5FNQ/ 4IFL	0.61/0.47	Brute force	1.3 billion	n.a.	69/590 (12%)	K_D_ = 114 nM	[39]
5-HT_2A_	5-HT_2A_: 7568 5-HT_2B_: 3616	5-HT_2A_: 12 5-HT_2B_: 97	homology model ^d^ 5TVN	0.67	Brute force, focused library	75 million (tetrahydro-pyridins)	n.a.	4/17 (24%)	K_I_ = 0.67 µM (→ EC_50_ = 41 nM)	[45]
PKA	2500	343	5N3J	0.55	X-ray fragment screening, synthon-based	208 thousand fragments as synthons, 2.7 billion	93/106 (88%)	30/75 ^e^ (40%)	K_I_ = 0.74 µM	[47]
CB_1_	10,090	4	5ZTY	0.96	Synthon-based	<0.1% of 11 billion, 600 thousand minimal synthons, 1.5 million	60/80 (75%)	21/60 (35%)	K_I_ = 0.28 µM (→ K_I_ = 0.9 nM)	[48]
ROCK1	3552	26	2ETR	0.53	Synthon-based	<0.1% of 11 billion, 600 thousand minimal synthons, 1 million superstructures	21/24 (88%)	6/21 (29%)	IC_50_ = 6.3 nM	[48]
ROCK1	3552	26	2ETR	0.53	Synthon-based	137 thousand fragment-sized building blocks, 5.2 million superstructures	n.a.	27/69 (39%)	K_I_ = 38 nM	[49]
SARS-CoV-2 M^pro^	201 (1765) [57]	774	6W63/ 5RF7	0.15/0.12	Brute force; focused (fragment)	235 million; 2 million	n.a.	19/100 (19%); 21/93 (23%) ^f^	K_D_ = 23 µM; K_D_ = 7.2 µM	[52]
SARS-CoV-2 M^pro^	201 (1765) [57]	774	4MDS	0.23	Brute force. deep learning	1.3 billion	0/0 n.a.	0/0 1/32 (3%)	0 IC_50_ = 0.8 mM ^g^	[53,54,55]
DNMT2	1 (16) ^h^	1	1G55	0.44	Filtering, brute force	720 million filtered to 3.4 million	18/21 (86%)	5/18 (28%)	K_D,app_ = 37 µM	This work
NSUN6	1 (5) ^h^	4	5WWR	0.33	Filtering, diversity subset	21.4 million filtered to 400 thousand, analog search in 14 billion	12/17 (71%)	5/12 (42%)	K_D,app_ = 12 µM	This work

^a^ According to ChEMBL (https://www.ebi.ac.uk/chembl/, accessed on 15 December 2022). ^b^ With 95% sequence identity to the entry used for docking (https://www.rcsb.org/, accessed on 15 December 2022). ^c^ Calculated with the DogSiteScorer [58,59] implementation of SeeSAR-12.0.1 for the PDB-ID used in the reported VS. Values between 0 and 1 with higher numbers indicating higher druggability. ^d^ The used template structure 5-HT_2B_ receptor (PDB-ID 5TVN) shares 67% sequence identity and 80% sequence similarity with the 5-HT_2A_ receptor. ^e^ 18 of 93 compounds were not sufficiently soluble for testing. ^f^ Hit rate in SPR binding assay. M^pro^ was inhibited by 3 and 5 compounds, respectively (hit rates of 3% and 5%). ^g^ In the original publication [55], no in vitro validation was performed. Re-scoring and testing was conducted by Rossetti et al. [53]. ^h^ Recently, 16 SAM-analog inhibitors of DNMT2 and 5 of NSUN6 that are not yet available in ChEMBL were discovered [25]. n.a.: Information on synthesis success rates are not available; D_4_, dopamine receptor type 4; AmpC, β-lactamase; MT1, melatonin receptor type 1; KEAP1, Kelch-like ECH-associated protein 1; 5-HT_2A_, serotonin receptor type 2A; PKA, protein kinase A; CB_1_, cannabinoid receptor type 1; SARS-CoV-2 M^pro^, severe acute respiratory syndrome coronavirus-2 main protease.

## 2. Results

### 2.1. Virtual Screening

**Figure 2:**
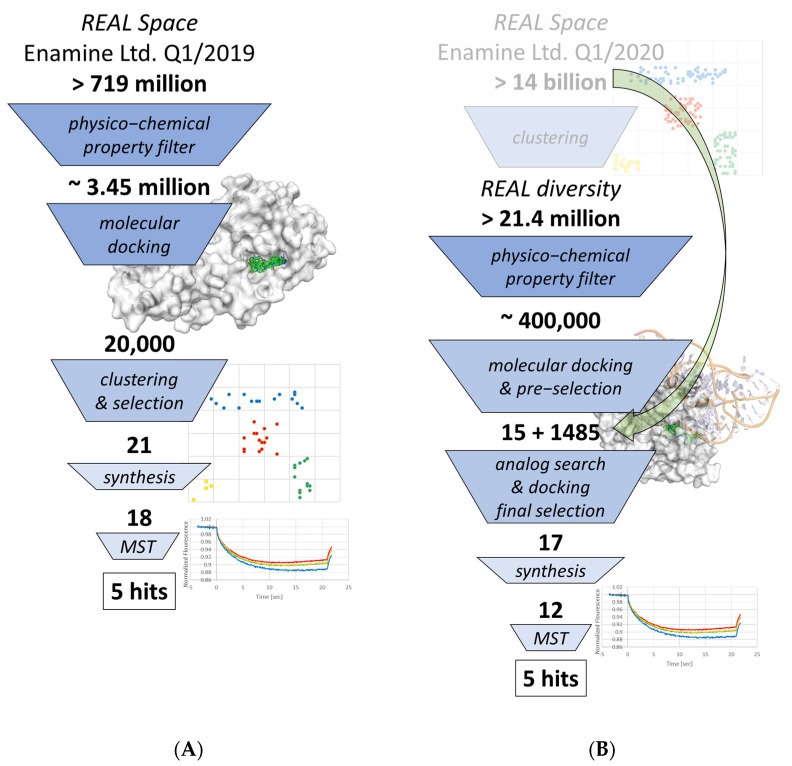
Virtual screening workflow for DNMT2 performing first molecular docking and then clustering (**A**), and NSUN6 starting from a diversity subset (first clustering) followed by docking and analog search in the whole chemical space (indicated by the green arrow) prior hit selection and testing (**B**).

In order to identify new chemotypes as DNMT2 and NSUN6 inhibitors distinct from the native ligand SAM, virtual screenings of the Enamine Ltd. readily accessible (REAL) chemical space were performed. For the DNMT2 virtual screening (Figure 2A), REAL Space consisted of 719,205,874 compounds, which was too large for a brute-force docking approach and required rather strict physicochemical property filtering (Table S1). Besides the removal of reactive or pan-assay interference compounds (PAINs) [60,61] and consideration of typical drug-like criteria according to the Lipinski rule of five (RO5) [33] and Oprea lead-likeness [62], additional truncation was performed based on the native ligand SAM, which is moderately large and very polar. By the application of upper and lower limits on molecular weight, rotatable bonds, charge, ring number and size, polar surface area (PSA), and chiral centers to reduce chemical complexity for future optimization, the screening library was reduced to a computationally feasible number. The remaining 3,447,976 molecules were docked against the DNMT2-SAH complex structure (PDB-ID 1G55) [63]. Among the 300 best-scoring compounds, which were visually inspected, several close analogs were observed. Subsequently, the top 20,000 molecules were also clustered prior to the final selection of 21 structurally diverse ligands from the top 300 clusters for testing (compounds **1.1**–**1.21**, Table S2). A total of 18 of these 21 were successfully synthesized by Enamine Ltd. (Kyiv, Ukraine) with the company’s robust internal procedures.

Differently from the DNMT2 virtual screening procedure (Figure 2B), instead of starting from the whole REAL Space (at the time over 14 billion molecules), the subset REAL diversity (Tanimoto similarity between compounds of less than 0.65 using the Morgan 2, 512 bit fingerprint according to Enamine Ltd.) of 21,441,180 compounds was subjected to physicochemical filtering (Table S1), resulting in only 400,306 molecules for docking against the NSUN6-SFG complex structure (PDB-ID 5WWR, tRNA present in the crystal structure was removed prior to docking) [64]. After visual inspection of the top 300 hits by docking score, for 15 selected compounds, 99 analogs per molecule were searched by structural similarity in the complete REAL Space and subsequently docked. Notably, only four analog series molecules with better scores compared to the initial hits were found. This and the overall lower faction of very high-scoring molecules hinted to the previously described hypothesis [36] that while hits are among the best of their respective clusters, other promising scaffolds with a worse scoring representative got lost during this process. The final hit selection consisted of nine initial hits from the diversity subset and four initial hit + analog pairs. Syntheses by Enamine Ltd. were successful for 12 of these 17 molecules (compounds **2.1**–**2.17**, Table S3).

**Figure 3:**
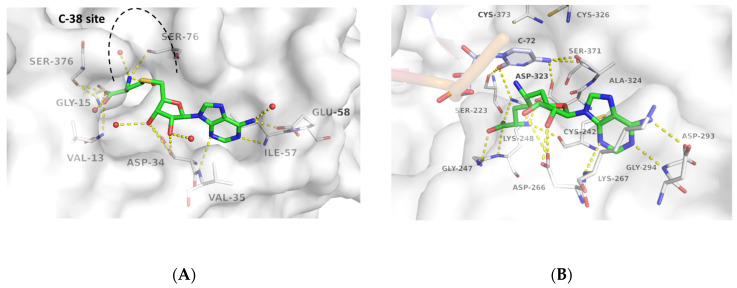
Binding modes of SAH-bound to DNMT2 (**A**) (PDB-ID 1G55) and SFG bound to NSUN6 (**B**) (PDB-ID 5WWR). Enzymes are shown with white surface and carbon atoms, ligands with green carbon atoms. Polar contacts are shown as yellow dashed lines, water molecules as red spheres. For clear view only residues forming polar contacts with the ligands are shown as lines and labeled as well as C-72 (light blue carbon atoms) and the catalytic Cys-residues 326 and 373 in the NSUN6-tRNA-SFG complex (**B**).

Even though the two virtual screenings were performed independently and separated in time, the hit selection criteria for both DNMT2 and NSUN6 were similarly based on the docking score as a first filtering step and the resembling of the crystallographic ligands’ interactions with the RNA methyltransferases. Special emphasis was put on molecules to not have peculiar internal torsion strain and being deeply burrowed in the SAM amino acid-moiety sub-pocket to result in H-bond interactions with the Gly-15 and Val-13 backbone as well as the Ser-376 sidechain in DNMT2 (Figure 3A) or Gly-245, Lys-248 backbone, and Ser-223 and additionally Lys-248 sidechain in NSUN6, respectively (Figure 3B). Followed by an eventually rigidified cyclic-aliphatic or aromatic linker that was allowed to enter the binding site of Cyt-38 in DNMT2 or Cyt-72 in NSUN6 (docking was performed without the tRNA present in the crystal structure), a mimic of the ribose vicinal diol interaction with Asp-34 or Asp-266, respectively, was prioritized. Lastly, substructures resembling the interactions of the adenine moiety of SAH and SFG, namely an H-bond donor to Glu-58/Asp-293, and H-bond acceptors for the backbone of Ile-57/Gly-294 and Val-35/Lys-267 (enumeration DNMT2/NSUN6) incorporated in or attached to an aromatic ring system, were favored.

### 2.2. Binding Assay and Structure–Affinity Relationship

Due to previous library filtering (Table S1), none of the obtained compounds was flagged as PAINs [61], potential aggregators, or reactive species. All virtual screening hits were subjected to a microscale thermophoresis (MST) pre-screening at three different concentrations of 300, 100, and 33.3 µM (Tables S2 and S3). MST proved to be especially suitable as a primary binding assay due to its high sensitivity and robustness that allowed the application for very weak binders or even fragments [65] and was demonstrated to be highly accurate for DNMT2 and NSUN6 ligand identification, previously [25,66]. For the literature known reference ligand SAH, K_D_ values of 11.8 µM and 9.1 µM for DNMT2 and NSUN6, respectively, were determined (Table 2). Virtual screening hits showing a dose-dependent shift of thermophoresis (Tables S2 and S3) were measured at additional concentrations to obtain K_D_ values. However, due to limited solubility, this was not always achieved when the upper plateau of MST dose–response curves could not be reached. Subsequently, apparent K_D_ values (K_D,app_) are presented when possible as a lower limit (indicated as K_D,app_ ≥ fitted value).

From the DNMT2 virtual screening, five hits could be identified as binders via MST (Table 2). The strongest binder of DNMT2 was **1.4** with K_D,app_ = 37 µM, while for **1.14** a K_D,app_ ≥ 67 µM could be determined. **1.6**, **1.17**, and **1.18** showed a reproducible, dose-dependent shift of thermophoresis in the dose–response curve and, thus, also binding. However, a K_D(app)_ value could not be determined clearly and is estimated to be in the high micromolar to millimolar range.

MST confirmed five ligands out of the NSUN6 virtual screening as well. K_D,app_ values of 16.4 µM, 42 µM, ≥72 µM, ≥83 µM and ≥369 µM could be determined for **2.4**, **2.8**, **2.5**, **2.2,** and **2.1**, respectively (Table 3).

**Figure 4:**
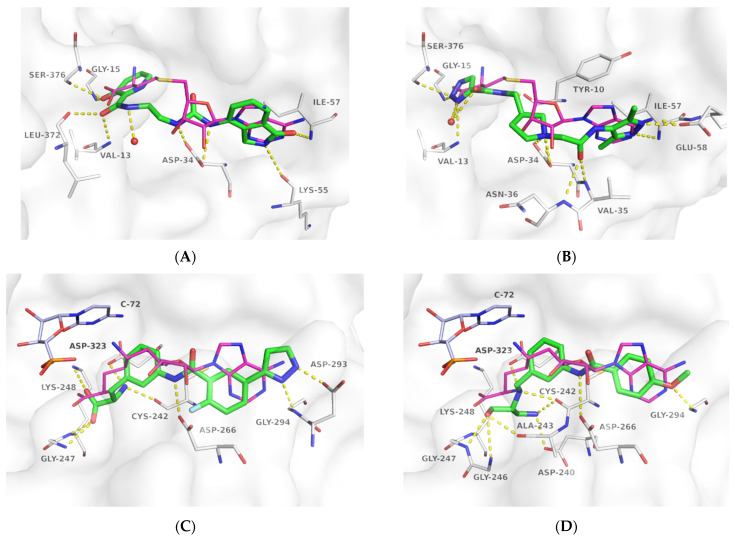
Predicted binding modes of **1.4** in complex with DNMT2 (**A**), **1.18** in complex with DNMT2 (**B**), **2.5** in complex with NSUN6 (**C**), and **2.8** in complex with NSUN6 (**D**). Docking poses are depicted with green carbon atoms, enzymes with white carbon atoms, and transparent surfaces. For a clear view, only residues forming polar interactions (yellow dashed lines) are shown and labeled. For orientation, the crystallographic reference ligands SAH (DNMT2, PDB-ID 1G55) and SFG (NSUN6, PDB-ID 5WWR) are shown with magenta carbon atoms. In NSUN6, C-72 is depicted with light blue carbon atoms for orientation, but tRNA was removed during molecular docking.

For DNMT2 hits (Table 2), regularly observed features in predicted binding modes were mimics of the H-bond acceptor profile of SAM’s methionine amino acid carboxylate sub-structure. While not necessarily being charged, for **1.4** (Figure 4A), instead of the docked and depicted protomer, a phenolate anion also seems reasonable due to the vinylogous acid with a predicted pK_a_ of 7.04 (calculated with MOE). More often, H-bond acceptors were found in a heterocycle-like triazine (**1.17** and **1.18**) or oxadiazole (**1.6**). A mimic of the native ligand’s basic, primary amine, however, was not found in the virtual screening hits. Connected by different types of linkers, the ribose hydroxy groups are replaced by either a urea (**1.4**, Figure 4A), an amide (**1.6**, **1.14**), or a basic nitrogen (**1.17**, **1.18**, Figure 4B) to interact with Asp-34. Lastly, the natural ligand’s adenine moiety and its H-bond interaction profile with Glu-58 and Ile-57 (Figure 3A) can be mimicked by an analog 4-amino quinazoline (**1.14**), an amide either attached to (**1.6**) or part of (**1.4**, Figure 4A) a ring system, or by a 3,5-dimethyl-1*H*-pyrazole (**1.17**, **1.18**, Figure 4B).

Differently from the DNMT2 hits where only mimetics of the acid were found, binders of NSUN6 feature the complete amino acid sub-structure (**2.2**, **2.5**, Figure 4C, Table 3) or a basic nitrogen alone (**2.1**, **2.4**), or even an additional positively charged group as in **2.8**, which interacts with Asp 240 (Figure 4D). In **2.2**, **2.5**, and **2.8**, a meta-substituted benzene linker was found as a common feature attached to an amide, which acts as an H-bond donor for Asp-266 replacing the interaction of one of the ribose hydroxyls according to the docking predictions (Figure 4C,D). As the adenine replacement, a variety of different one- or two-ring systems was found.

One intention of the virtual screening was the identification of novel chemotypes distinct from the native ligand SAM and eventually improved selectivity for the target RNA methyltransferase. Even though there are some differences in sequence identity and similarity within the SAM-binding sites (17% identity, 31% similarity), interaction profiles are highly conserved between DNMT2 and NSUN6 (Figure 3A,B). Testing of hits from the DNMT2 virtual screening against NSUN6 and vice versa, however, showed selectivity for **1.6**, **1.17**, **1.18** for DNMT2 and **2.4**, **2.5**, and **2.8** for NSUN6 as intended (Table 2 and Table 3, right column). Molecules were defined to be selective when no MST shift was observed for the other (‘off-target’) enzyme at a ligand concentration of up to 300 µM. Notably, non-selective NSUN6 ligands **2.1** and **2.2** (Table 3) contain an amino acid or only the basic moiety, a feature not found in the DNMT2 virtual screening. This indicates that the presence of the basic nitrogen is underestimated in the DNMT2 docking, which was observed previously when for SAM-analog DNMT2 inhibitors, a drastic loss of potency was observed upon removal of the positively charged nitrogen from a SAH-scaffold [25]. It was hypothesized that this basic amine is involved in an H-bond network with several water molecules not captured by the docking protocol. Further, **1.4** (DNMT2 K_D,app_ = 37 µM) turned out to be the strongest binder of NSUN6 (K_D,app_ = 12 µM) even though derived from the DNMT2- and not the NSUN6-virtual screening. Likewise, **1.14** was not selective over NSUN6 with a K_D,app_ ≥ 116 µM (DNMT2 K_D,app_ ≥ 67 µM), and for **2.2** from the NSUN6 docking, a DNMT2 K_D,app_ ≥ 145 µM was determined.

Lastly, compounds **1.4**, **1.6**, **1.14**, **1.17,** and **1.18** were subjected to a DNMT2 tritium incorporation activity assay at a concentration of 100 µM. However, the low binding affinity in the mid-micromolar to presumably millimolar range did not effectively translate into significant enzyme inhibition (Figure S1). Based on crystal structure analysis, there is no evidence for an allosteric druggable binding site. Eventually, the presence of substrate tRNA might induce conformational changes in the catalytic loop [67] of the enzyme-altering ligand binding strength and behavior compared to the tRNA-free MST binding assay. Another hypothesis, even though rather speculative and to be taken with caution, is that the free energy of ligand binding is spent to ‘flip-out’ C-38 of tRNA^Asp^ for methylation [68], forming a more stable ternary DNMT2-tRNA-inhibitor complex compared to the DNMT2-inhibitor complex alone. However, the required structural, thermodynamic, and, eventually, kinetic characterization of these complex formations is beyond the scope of this manuscript and likely requires more potent ligands for in-depth elucidation.

## 3. Discussion

The advance of commercial chemical spaces allows virtual screenings of a novel yet synthetically accessible chemical matter for targets of interest. Applying two different strategies of chemical space docking screens led to the identification of novel binders with K_D,app_ values down to 12 µM for RNA methyltransferases that have low predicted druggability, DNMT2 and NSUN6 (Table 2 and Table 3). While for both targets, the virtual screening strategy included strict physicochemical property filtering (Table S1) based on known ligands’ parameters to reduce the library size to a computationally feasible number of molecules, different methods of diversification were applied (Figure 2). While for DNMT2, a larger library of 3.4 million molecules was docked and clustered afterward (‘first dock, then cluster’), for NSUN6, a diversity subset was used as a starting point for filtering and docking followed by an analog search (‘first cluster, then dock’). Interestingly, hit numbers were similar for both strategies, with 5 of 18 for DNMT2 and 5 of 12 for NSUN6, respectively, even though it is hypothesized that diversity subsets might lose complete clusters of potential ligands if the cluster representatives do not resemble favorable interaction profiles [36]. This was indirectly hinted during the hit selection process when initial virtual screening hits from the REAL diversity subset for NSUN6 usually showed lower scores than their analogs from REAL Space (Table S3). However, handling the increasing size of commercial (and proprietary) [41] chemical spaces will require new strategies to focus libraries prior to computationally more expensive molecular docking screens [43,44,45] or improvements in fragment docking and scoring [50,51] to enhance synthon-based chemical space design within the binding site [47,48,49]. In this study, both DNMT2 and NSUN6 binders showed affinities in the mid-micromolar to presumably millimolar range in an MST binding assay. **1.4** and **1.14** were the strongest identified binders of DNMT2 with K_D,app_ = 37 µM and ≥67 µM, respectively. For NSUN6, the highest affinity was found for **2.4** (K_D,app_ = 16.4 µM) and **2.8** (K_D,app_ = 42 µM). However, selectivity between the two methyltransferases was not always achieved, as seen, for example, in compound **1.4,** which originates from the DNMT2 virtual screening but is also the strongest binder of NSUN6 with a K_D,app_ of 12 µM. Hence, the identified compounds can be considered initial hits as starting points for further hit-to-lead optimization. While no analogs of these novel chemotypes are available in commercial in-stock libraries, their origin from REAL Space still allows fast and easy derivatization either by the combination of the available building blocks or a direct SAR-by-catalog approach to improving the inhibitory potency and selectivity for the development of DNMT2 and NSUN6 ABPs in the future.

## 4. Materials and Methods

### 4.1. Virtual Screening

The virtual, combinatoric synthesis molecule libraries REAL Space and REAL diversity were obtained from the supplier’s homepage (Enamine Ltd., https://enamine.net/compound-collections/real-compounds/ accessed on 14 January 2019 for REAL Space and 10 August 2020 for REAL diversity) in SMILES format. Physicochemical property-filters (Table S1) to reduce library sizes were applied with MOE (Molecular Operating Environment (MOE), 2018.0101 Chemical Computing Group ULC, 1010 Sherbooke St. West, Suite #910, Montreal, QC, Canada, H3A 2R7, 2018.) and FILTER (FILTER part of OMEGA 3.1.0.3: OpenEye Scientific Software, Santa Fe, NM, USA, http://www.eyesopen.com, 2018). Energetically favorable 3D conformers for docking were generated using OMEGA [69].

For DNMT2 virtual screening, the DNMT2-SAH complex structure (PDB-ID 1G55) [63] and the FlexX-3.0 [70] (BiosolveIT GmbH. FlexX v.3.0 Sankt Augustin, Germany, 2018) as the docking engine were used. The docking setup was validated by re-docking of the crystallographic reference ligand SAH (FlexX-score: −38.08 kJ/mol, RMSD: 0.997 Å). The filtered 3.45 million compound library derived from REAL Space was docked under these conditions. Top-scoring molecules of rank 1–300 were visually inspected for hit selection. Additionally, the top 20,000 molecules were clustered using MACCS fingerprints and the Tanimoto coefficient similarity metric (max. 0.65) within MOE. The top 300 clusters were also considered during hit selection for testing (Table S2).

For docking setup validation of the NSUN6 virtual screening with FlexX-4.1 (BiosolveIT GmbH. FlexX v.4.1 Sankt Augustin, Germany, 2019), re- and cross-docking of SAM and SFG from PDB-IDs 5WWR and 5WWS were performed for chains A and B in presence and absence of the tRNA present in the crystal structure, respectively [64]. Additionally, scoring was evaluated by docking SAM, SFG, and SAH and 150 decoys derived from the database of useful decoys-enhanced (DUD-E) [71] with similar physicochemical properties but distinct structural features. Even though FlexX is suitable for RNA-ligand docking [72], a docking setup without the tRNA present in the crystal structure was selected to allow potential ligands to not only bind to the SAM-, but also the Cyt-72 sub-pocket as demonstrated previously [25]. Additional interactions within this site hold the potential of improved binding affinity and selectivity while also interfering with tRNA binding. The NSUN6 docking screen was subsequently performed with PDB-ID 5WWR, chain B, which showed reasonable posing and scoring for SFG re-docking (FlexX-score: −43.63 kJ/mol, rank: 2/153, RMSD: 1.09 Å), SAM cross-docking (FlexX-score: −46.32 kJ/mol, rank: 1/153, RMSD: 0.93 Å) and SAH docking (FlexX-score: −36.71 kJ/mol, rank: 18/153). Docking of the filtered REAL diversity library of around 400,000 molecules was performed under the same conditions. After visual inspection of the 300 top-scoring molecules, analogs of 15 molecules were searched in the complete REAL Space (at the time over 14 billion molecules) using infiniSee-1.2 [73,74] (BiosolveIT GmbH. infiniSee v.1.2 Sankt Augustin, Germany, 2019) and 99 analogs for each of the 15 initial molecules were also docked prior final compound selection for testing.

Molecules were ordered from Enamine Ltd. Custom synthesis based on the company’s internal procedures was successful for 18 out of 21 compounds from the DNMT2 virtual screening (86% synthesis success rate) and 12 of 17 for the NSUN6 virtual screening (71% synthesis success rate), respectively. Identity and purity > 90% of obtained compounds were guaranteed by the supplier and confirmed for MST hits using in-house LC/ESI-MS analysis (Tables S2 and S3). HPLC/ESI-MS analysis was performed using an Agilent 1100 series HPLC system with an Agilent Poroshell 120 EC-C_18_ (150 × 2.10 mm) or an Agilent Zorbax SB-Aq (4.6 × 150 mm) column (both at 40 °C oven temperature) with MeCN/H_2_O + 0.1% HCOOH = 10:90 → 100:0 as a mobile phase at a flow rate of 0.7 mL/min. Samples were applied using 5 μL injection with quantitation by AUC at 254 nm or 210 nm. Electrospray ionization (ESI) mass spectra were recorded on an Agilent 1100 series LC/MSD Ion trap spectrometer in the positive ion mode.

Figures are made with PyMOL (The PyMOL Molecular Graphics System, Version 2.4.0 Schrödinger, LLC.). The background of the TOC figure was generated with craiyon (https://www.craiyon.com/ accessed on 15 December 2022).

### 4.2. Protein Expression and Purification

The plasmid containing genes for DNMT2 was kindly provided by Albert Jeltsch (University of Stuttgart, Stuttgart, Germany). Expression and purification were performed as described previously with minor adaptions [25]. In brief, the concentration of sodium chloride in the buffers used for immobilized metal affinity chromatography was increased to 500 mM to remove more unspecific bound impurities from DNMT2; therefore, ion-exchange chromatography was skipped. A plasmid for the expression of NSUN6 was designed and synthesized as described previously [25] (made available via Addgene, ID: #188060, https://www.addgene.org). Expression and purification were performed according to the literature. Plasmids coding for each enzyme were separately transformed into *E. coli* Rosetta2 cells. These were grown in LB medium at 30 °C overnight. The next day, 1 L TB medium was inoculated with 20 mL overnight culture. Cells were grown at 37 °C until an OD_600_ of ~0.8 was reached, then the temperature was reduced to 20 °C for DNMT2 and 16 °C for NSUN6, respectively. Overexpression of the proteins was induced by adding isopropyl-β-D-thiogalactopyranoside (IPTG) to a final concentration of 500 µM. Overexpression was maintained overnight. Cells were harvested by centrifugation. After cell lysis by sonication, cell debris was removed by centrifugation, and the supernatant was objected to immobilized metal affinity chromatography (Ni^2+^-NTA, HisTrap HP, 5 mL) for further purification a size-exclusion chromatography (Superdex 16/600 75 PG) was performed using an ÄKTA Start (GE Healthcare, Chicago, IL, USA). Glycerol concentrations were adjusted to allow liquid storage of proteins at −20 °C until further use.

### 4.3. Microscale Thermophoresis

Since the constructs of DNMT2 and NSUN6 contain hexa-histidine tags, proteins were labeled using a Monolith His-Tag Labeling Kit RED-Tris-NTA 2nd generation according to the manufacturer’s instructions. This labeling strategy was chosen since it should prevent any interference with the actual binding site of the proteins. Labeled protein was diluted to a concentration of 20 nM into MST buffer (50 mM Hepes, pH 7.5, 150 mM NaCl, 10 mM MgCl_2_, 1 mM DTT, 0.05% polysorbate-20, 0.1% PEG-8000). All compounds were prepared as stocks dissolved in DMSO to a concentration of 50 mM. For all compounds, dilutions in MST buffer to concentrations of 600 µM, 200 µM, and 66.7 µM, respectively, were prepared. Labeled protein was then mixed 1:1 with the dilution series of each compound (final concentrations: 10 nM protein, ligands 300, 100, and 33.3 µM, respectively) and incubated for 5 min at room temperature. All measurements were performed on a Monolith Pico instrument (NanoTemper Technologies, Muenchen, Germany) with red light. To induce thermophoresis, medium MST power was selected for DNMT2 and high MST power for NSUN6. All experiments were performed in quadruplicates. For all compounds that showed a concentration-dependent thermophoresis behavior, a half-logarithmic dilution series was prepared to cover a range from 600 µM to 600 nM. Obtained dilutions were then mixed 1:1 with labeled protein (20 nM) and incubated for 5 min at room temperature prior to measurement. Experiments were performed in duplicates. All data received were analyzed using the MO. Affinity Analysis software version 2.3 (NanoTemper Technologies, Muenchen, Germany).

### 4.4. Tritium Incorporation Assay

DNMT2 activity assays were carried out in 20 µL containing 100 mM Tris-HCl, pH 8, 100 mM NH_4_OAc, 0.1 mM EDTA, 10 mM MgCl_2,_ and 10 mM DTT. The amount of DMSO in the reaction mixture was adjusted to 5%, while tRNA^Asp^ was added to a final concentration of 5 µM after heating it to 75 °C for 5 min and slowly cooling it to room temperature. To this, SAM was added as a mixture of cold SAM (New England Biolabs GmbH, Ipswich, MA, USA) and ^3^H-SAM (Hartmann Analytics, Braunschweig, Germany) to final concentrations of 0.9 µM and 0.025 µCi µL^−1^. DNMT2 was added last to a concentration of 250 nM, and enzymatic reactions were run at 37 °C. Aliquots of 8 µL were taken out of the reaction mixture at 0 and 20 min, spotted on Whatman^®^ glass microfiber filters (GF/C, 25 mm), and transferred into an ice-cold trichloroacetic acid (TCA) solution (5%) where they were kept for at least 15 min. Subsequently, two washing steps with the TCA solution (5%) for 20 and 10 min and one with EtOH for 10 min were carried out at room temperature. The filters were dried and placed into scintillation vials. A total of 3 mL of Gold MV liquid scintillation cocktail (PerkinElmer, Waltham, MA, USA) was added before scintillation was measured for 1 min on a scintillation counter (TriCarb^®^ Liquid Scintillation Analyzer 4810TR, PerkinElmer, Waltham, MA, USA). For the inhibition assay, compounds were present at a final concentration of 100 µM during the enzymatic reaction, and inhibition in percent was calculated by referencing the scintillation signal to a positive control without compound. All experiments were carried out in biological triplicates, while errors refer to the obtained standard deviation.

## Figures and Tables

**Table 2:** MST results of SAH and newly identified DNMT2-ligands derived from the DNMT2 virtual screening. Measured normalized fluorescence values (Fnorm [‰]) are mean with standard error of at least duplicate determination. Apparent K_D_ values (K_D,app_) are indicated as a lower limit (via the ≥ symbol) if the upper plateau of the dose–response curve was not completely reached. In case a dose-dependent shift in thermophoresis was observed, which indicates binding, but the curve could not be fitted with sufficient accuracy (**1.6**, **1.17**, **1.18**), K_D,app_ was not determined (n.d.). Molecules are depicted in their docked stereoisomers, protomers, and tautomers; however, for **1.6**, **1.17**, and **1.18**, racemic mixtures were obtained for testing. All molecules are drawn in the same orientation as SAH, with left side: amino acid mimetic, central part: ribose replacement, right side: adenine mimetic.

Compound	MST Dose–Response Curves	
	DNMT2 (Primary Target)	NSUN6 (‘Off-Target’/Selectivity)
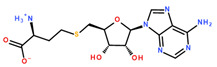 SAH	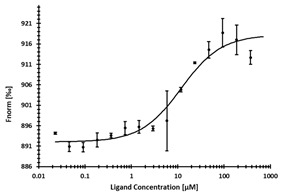 K_D_ = 11.8 µM	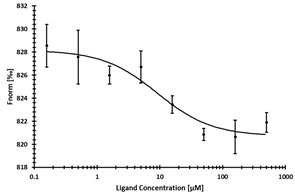 K_D_ = 9.1 µM
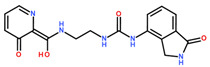 **1.4**	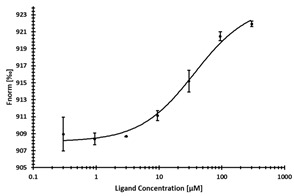 K_D,app_ = 37 µM	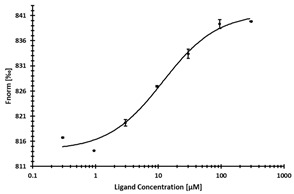 K_D,app_ = 12 µM
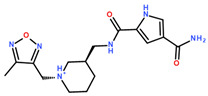 **1.6**	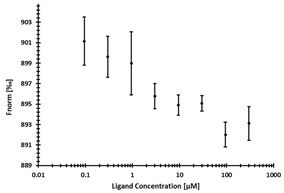 K_D,app_ = n.d.	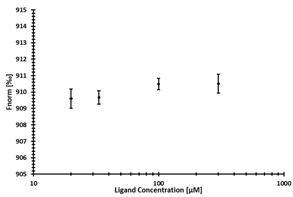 No binding
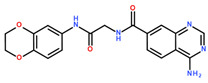 **1.14**	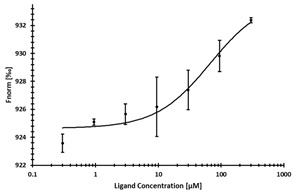 K_D,app_ ≥ 67 µM	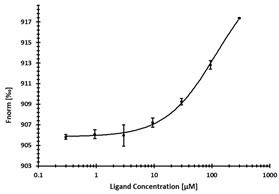 K_D,app_ ≥ 116 µM
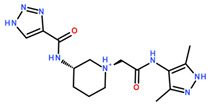 **1.17**	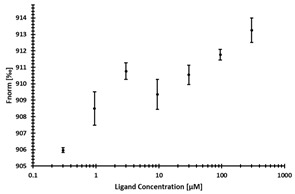 K_D,app_ = n.d.	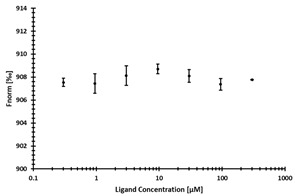 No binding
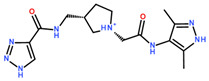 **1.18**	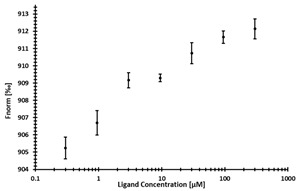 K_D,app_ = n.d.	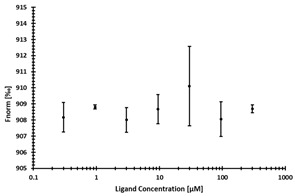 No binding

**Table 3:** MST results of newly identified NSUN6-ligands derived from the NSUN6 virtual screening. Measured normalized fluorescence values (Fnorm [‰]) are mean with standard error of at least duplicate determination. Apparent K_D_ values (K_D,app_) are indicated as a lower limit (via the ≥ symbol) if the upper plateau of the dose–response curve was not completely reached. In case a dose-dependent shift in thermophoresis was observed, which indicates binding, but the curve could not be fitted with sufficient accuracy, K_D,app_ was not determined (n.d.). Molecules are depicted in their docked stereoisomers, protomers, and tautomers; however, for **2.1** and **2.4**, racemic mixtures were obtained for testing. All molecules are drawn in the same orientation as SAH, with left side: amino acid mimetic, central part: ribose replacement, right side: adenine mimetic.

Compound	MST Results	
	NSUN6 (Primary Target)	DNMT2 (‘Off-Target’/Selectivity)
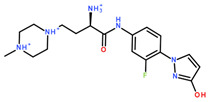 **2.1**	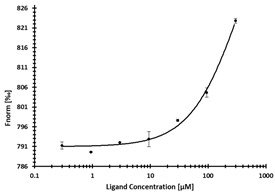 K_D,app_ ≥ 369 µM	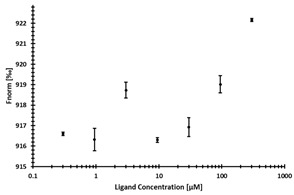 K_D,app_ = n.d.
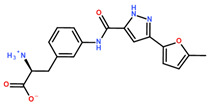 **2.2**	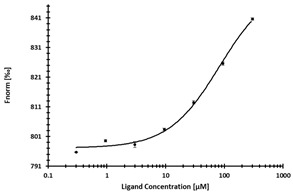 K_D,app_ ≥ 83 µM	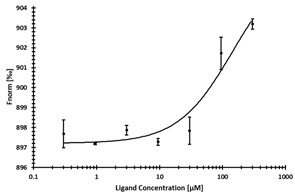 K_D,app_ ≥ 145 µM
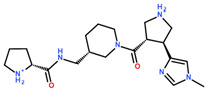 **2.4**	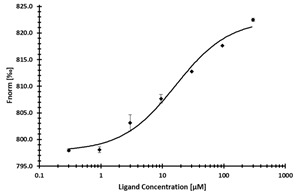 K_D,app_ = 16.4 µM	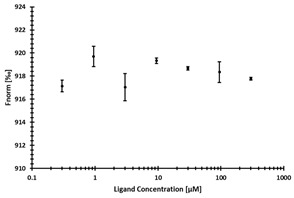 No binding
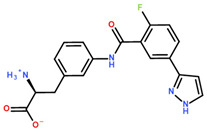 **2.5**	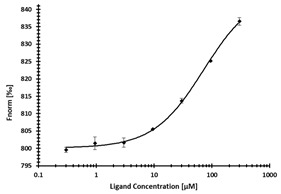 K_D,app_ ≥ 72 µM	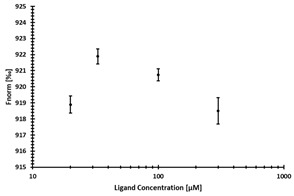 No binding
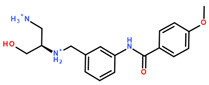 **2.8**	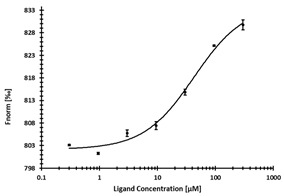 K_D,app_ = 42 µM	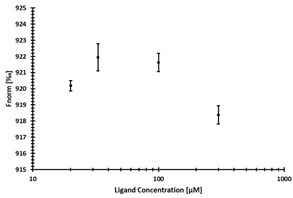 No binding

## Data Availability

Not applicable.

## Supplementary Materials

The following supporting information can be downloaded at: https://www.mdpi.com/article/10.3390/ijms24076109/s1. References [33,61,62,75] are cited in Supplementary Materials.

## Abbreviations

ABP, activity-based probe; AML, acute myeloid leukemia; AmpC, β-lactamase; CB_1_, cannabinoid receptor type 1; D_4_, dopamine receptor type 4; DNMT2, RNA methyltransferase 2; GPCR, G-protein-coupled receptor; 5-HT2A, serotonin receptor type 2A; HTS, high-throughput screening; IPTG, isopropyl-β-D-thiogalactopyranoside; KEAP1, Kelch-like ECH-associated protein 1; m5C, 5-methylcytosine; METTL3, methyltransferase 3; ML, machine learning; MST, microscale thermophoresis; MT1, melatonin receptor type 1; NSUN6, Nol1/Nop2/SUN 6; PDB, protein data bank; PKA, protein kinase A; PSA, polar surface area; REAL, readily accessible; SAH, S-adenosyl homocysteine; SAM, S-adenosyl methionine; SARS-CoV-2 Mpro, severe acute respiratory syndrome coronavirus-2 main protease; SFG, sinefungin; SMILES, simplified molecular input line entry specification; TCA, trichloroacetic acid.
